# Editorial

**Published:** 1855-01

**Authors:** 


### THE MEDICAL EXAMINER.

PHILADELPHIA, JANUARY, 1855.

### SALUTATORY.

With this number opens the eighteenth year of the Examiner's existence, and the second of our duties as its Editor; duties which were assumed with diffidence and mistrust of success, but with the earnest desire to do all in our power to render the journal a welcome guest to its readers. In this object of our wishes we trust that we have not altogether failed, and at this season, rife as it always should be with high aspirations and good resolutions, we indulge the hope that this year's labors may earn for it even a greater value than it has already long enjoyed. We gratefully acknowledge our indebtedness to those gentlemen who have so largely enhanced the interest and usefulness of its pages by their valuable contributions, and respectfully solicit from them and others a continuance of such support.

---

### CLINICAL INSTRUCTION.*

Of all the preliminary branches of medical education requisite to fit the student for his future duties as a practitioner, none should sustain a

*We had the pleasure, after writing the above, of hearing the very able and eloquent address of the President of the Philadelphia County Medical Society, Dr. Thos. F. Betton. In connection with other matters considered very reprehensible by Dr. B., the College clinics were severely commented on; our readers will do well to procure a copy of it.—Editor.

higher rank, in his estimation, than that of clinical or bed-side instruction. It is the great instrument for training him to future usefulness. To be able to recognize the different aspects of disease, to know when interference is unnecessary, if not hurtful, to know how to wield the various appliances and resources which science has so amply placed in his hands, are qualifications which he can only acquire through attentive observation, patient industry, and practical familiarity with disease. No lectures, however able, no amount of reading, however great, can ever supply the place of such experience to him. He may, it is true, when transformed by his diploma into a physican, go forth to his calling without such knowledge; but if he have a right sense of the awful and sacred responsibilities entrusted to him, and be actuated only by such motives as should influence the honest practitioner, how humiliating will frequently be the sense of his own incompetency, how deep and sincere his regret that he was denied or made no use of such advantages during his student life.

There are two systems now in operation in our large cities for affording information to the student of medicine—Hospital clinics and College clinics. The student who takes the hospital ticket, visits it twice weekly, the two hours of each visit being equally divided between surgery and the practice of medicine; where the class is too large to be admitted into the wards, lectures and operations take place, instead, in its amphitheatre, such cases as are required by the physician or surgeon being successively brought in there. It is much to be regretted that the large size of classes in this country renders it generally necessary to give this instruction in the amphitheatre, rather than in the wards. It must also be allowed that Hospital instruction bears too small a proportion to didactic teaching. Insufficient, however, as such instruction is, it possesses advantages which can never be obtained by any other mode, the student being afforded by it the opportunity of seeing *acute* cases of disease, fevers, phlegmasiæ and recent accidents, the staple of what his future practice will mostly consist; of following a case until recovery or death takes place, and in the latter instance, of having the advantages of the autopsy.

The other plan of clinical instruction, which, in comparison with the one just described, may be termed the modern system, has also two hours devoted twice weekly, in the College edifice, for the same purpose. The difference between the two being that, in this latter instance, the medical cases are generally chronic, reside at their own homes, and may or may not call for future advice. Such cases as require surgical interference are operated on before the class; and if severe, are then removed, either to rooms in the building, or else to some contiguous house. We would here observe, that the students of some institutions avail themselves of both of these plans of instruction. Where there is no requirement, however, for a hospital certificate for graduation, as is the case with some Colleges, but few of their pupils attend the hospital, the great majority naturally contenting themselves with their own College clinic.

The objections just urged against the hospital plan—excessive size of the class and infrequent attendance—are equally applicable to the College plan of clinical instruction. In addition to these objections, there are other evils appertaining to it, sufficient, we think, to justify the alarm, if they do not deserve the reprobation, of the whole profession. The nature of these evils, which pertain chiefly to the surgical clinic, and from which the student, the patient and the character of the profession are all liable to suffer, we shall now briefly discuss.

It is, we believe, acknowledged that nearly all students prefer the practice of surgery to that of medicine. Its points of interest are more prominent, it appeals more to the senses, and calls less upon the reflective faculties, than do the invisible, complicated and often obscure operations of internal disease. It is also more exciting. The polished and glittering knife, the well-filled tray, the resolute and self-reliant operator, the prostrate patient with his cries of agony and flowing blood, and finally the triumphant result, present a tableau, which, repugnant though it may be, has a terrible fascination for the young beholder. Is there no fear, that, in the excessive prominence and frequency of these displays, operative surgery, the most subordinate of all the branches of that science, may obtain an importance in the minds of students to which it has no legitimate claim; which it has been, in fact, the hope and study of modern science to prevent? Do not such exhibitions cultivate a taste for the knife, to the exclusion of more correct surgical principles? May not their frequent exhibition, also, render the tender mind of the young student callous and indifferent to the sufferings of his fellow creatures? Is not, in fine, the noble science of surgery degraded, rather than elevated, by the undue importance attached to such displays?

These are questions which we shall leave to the hearts and understandings of our readers. We would not be understood as denying that some information may be obtained by even distant inspection of operations. The question is, whether these, when placed in the foreground of clinical instruction, are not calculated to produce most erroneous impressions upon the mind of the students. The student is not, however, the only sufferer by this system; it is very possible that the patient may, also, be injuriously affected by it. Is there no temptation, for instance, for the surgeon to operate immediately whether the subject is in a suitable condition or not, for fear that some rival establishment may get the case ; or when such interference is not feared, and the patient is secured, that he may sometimes be kept waiting a long time, and until a more favorable opportunity for display may have arrived? We have heard of such instances. May not the natural desire, also, to render the clinic interesting, induce the surgeon to perform operations, which in his private practice he might consider unadvisable? Professors are not infallible. They are but men, with judgments very apt to be influenced by their interests; quite as prone, also, when running a race of competition, to forget everything but the excitement of the chase.

Serious as are the objections we have just stated, sufficient in themselves alone to condemn the whole system, they are as nothing in comparison with the injurious effects exerted and reflected by it upon the character of the profession at large. When those who hold the keys of our temples, the arch-dignitaries of our noble calling, to whom are entrusted the high responsibilities of educating the characters as well as the minds of their pupils, whose honors should be like Cæsar's wife, above even suspicion, descend into the arena with tricksters and charlatans by advertising their lists of cases, treated and operated on in their several institutions, by performing operations gratuitously upon those who have no claim for such charity; by offering inducements, direct or indirect, to influence patients to submit to the knife, which they would be ashamed to make use of in their private practice, and by setting in motion every available means to obtain cases for their clinics; can it be expected that the lesser lights of the profession should preserve their honor and integrity unsullied? Will they not, also, vaunt the number of *their* cases, the magnitude of *their* cures? May they not, endorsed by such high authorities, employ dishonorable and improper means to obtain notoriety and practice, and plead before the bar of conscience, the examples set before them? Is it a wonder that charlatanism should prevail throughout the land, or that we should be gradually sinking in our own and the community's estimation? Is there no fear, have we no right to fear such consequences?

Another serious and frequently urged complaint against this system, is that it almost entirely precludes the young practitioner from obtaining that portion of operative surgery, which would otherwise legitimately fall to his share. It is but natural, that the poor and persons in moderate circumstances should prefer the services of him whose reputation and abilities are well known, to those of any young aspirant, however able and well prepared. And thus the veteran professor, receiving his pay by the clinical exhibition of the case, virtually and in reality underbids his younger rival. We do not mean, of course, to detract from the merit of services gratuitously rendered from motives of charity, but the case is widely different when the surgeon receives his remuneration not exactly, it may be, in money, but in a coin perhaps even more valuable to him. The class of cases to which we have alluded, would not, under other circumstances, compensate the surgeon of eminence for the time he would be obliged to devote to them. Under the existing state of things, however, the young man who is desirous of obtaining a reasonable share of this practice, discovers that the competitor who enters the lists with the *most* ardor against him, and who not only accepts willingly all such cases, but who leaves no stone unturned to obtain them, is actually at the very head of the profession. Such monopoly, we need not say, effectually closes the door against the young practitioner.

Such are the legitimate fruits of the present system of College clinics; alike mischievous to the student and discreditable to the profession. The question is, what is to be done under the circumstances? In laying down any scheme of medical education some regard should be paid to the established usages and forms which the necessities of our position have forced upon us. Our requirements should not, therefore, be utopian. Let us return to the old-fashioned, but respectable plan of hospital instruction. Let every institution in the country, empowered to grant a degree, demand a hospital certificate of its pupils. Let the hours now devoted to the College clinic be added to those already given at the hospital, thus doubling the opportunities there afforded the student, without any additional loss of his time of attendance upon lectures, and finally, let two hours be set apart weekly to witness post-mortem examinations. Let these changes take the place of the present disgraceful and incompetent plan, and we have every reason to believe that the reform will be an advantageous one to the whole profession. We distinctly disclaim the imputation that our remarks are directed against either particular institutions or individuals. They have a wider scope. As regards the Faculties of our own city, we can bear willing and cheerful testimony to their general high character. Among them are many gentlemen whose attainments, elevated professional standing, courteous demeanorand noble liberality have won for them the respect and love of all who know them. As lecturers, also, in their several branches, we believe that, as a class, they are unsurpassed anywhere. These circumstances, however, but add to our regret that we cannot exempt any of them from the stigma which we think to be deservedly attached to every one who is connected with this system. They should reflect that no plea of self-preservation, no desire to outstrip a competitor, can ever absolve them from their responsibilities for the proper use of the powers entrusted to them. Would we could say that they duly felt their obligations. Would that we could say that the bright legacy of confidence and respect bequeathed them by their predecessors was still untarnished.

We have dwelt at some length upon this matter, as we thiuk it is one connected not only with the rights of students, but with the honor of the profession. We shall not pause to enquire into the motives which prompted the introduction of the College clinic, nor upon whom the odium of its origination rests; neither shall we attempt to investigate the degrees of criminality of different Institutions in this matter, but shall merely add, that, did we believe that it was a necessary evil, the result of circumstances over which there could be no control, we should have spared ourselves the trouble of making these remarks. When we shall have heard that any appeal has been made to the managers of our numerous hospitals, or that any other measures whatever have been taken to increase the present means of accommodation for the student, then, and not till then, will we believe that the College clinic is a necessary evil, and not a mere advertisement,—whose chief results have been to dazzle and ensnare the medical student.

The following extract from "The Address to Students," by the editor of the London Medical Times and Gazette, shows the high importance attached abroad to Hospital Instructions. We commend it to the reflection of our readers.

"The question which, after entry on Hospital studies, will most engage the attention of the zealous student will be as to the relative amount of time to be devoted to the wards, to the lecture-room, and to private reading. On this point, the regulations of the Examining Boards are explicit, requiring certificates of attendance on practice during the whole period of study. An opinion fraught with the most mischievous consequences has, however, got about, in favor of a division of the period, and the devotion of the latter part only to the acquisition of practical knowledge. It is customary to hear that 'the first year's man is not expected to attend Hospital practice,' 'that he has enough to do with his lectures,' etc. Unless they are intending to devote to educational purposes a much longer series of years than are usually so spent, we cannot do otherwise than urge upon our young friends not to listen for one moment to such advice. Out of the three or four years within which residence at a Medical school is ordinarily comprised, not a single day should be allowed to pass without some hours of it being devoted to attendance in the wards. It is there only that the student will learn to estimate duly the relative importance of the different subjects for which his attention is asked; it is there alone the real fitness for the duties of after-life is to be obtained. The published views of Dr. Latham are well known. We quote the following from no less an authority than Dr. Graves:—'Five or six years' attendance on an Hospital will be little enough to qualify you to enter with propriety and confidence on the discharge of your Professional duties. Bear in mind, gentlemen, that when you come to treat diseases, you approach the bedside as Physicians or Surgeons, and not as chemists, botanists or anatomists. This is the character in which you are to appear; and to the acquisition of knowledge which will prepare you for the discharge of its duties you ought to apply your chief attention.' In another part of the same lecture Dr. Graves adds:—'I would not be understood to depreciate any department of human knowledge. Far be it from me; besides the attempt would be useless. But I am anxious that you should concentrate all your energies on the proper objects of Medical pursuit, and devote the largest share of your attention to those acquirements which render you good Practitioners.'' The student may probably object to the recommendation to begin Hospital practice at once, that, without a knowledge of anatomy and physiology, he is at a loss to understand the diseases which come before him; and that, while ignorant of the principles of therapeutics, the cures he sees made teach him no useful lessons. To this we answer, that the attempt to supply knowledge before the need of it has been felt is always attended by much waste of labor. It is far better, for instance, that, coincidently with the acquisition of anatomical details, the practical importance of those details should be strongly felt, than that they should be esteemed only as dry, unproductive facts. The memory requires all the assistance which association of ideas can be made to give it. Who has not experienced how faithful has been his retention of any piece of knowledge for which he had long sought, or which was supplied just at a juncture when he was capable of perceiving its full weight? Just so it is with the light afforded by anatomy and physiology to him who in his daily study of disease is puzzled for the want of it. But, setting aside the advantage thus gained in respect to the aptitude for learning these sciences themselves, the experience accumulated, the training of the perceptive powers, and the acquisition of tact, which are consequent on constant Clinical work, will abundantly reward for its prosecution, even under some disadvantage as to imperfect knowledge of preliminaries.

